# Management of concurrent severe COVID-19 pneumonia and antibody-mediated rejection following kidney transplantation: a case report

**DOI:** 10.3389/fmed.2025.1521785

**Published:** 2025-03-13

**Authors:** Qiuxiang Xia, Heng Li, Kailun Sun, Hanying Li, Xianpeng Zeng

**Affiliations:** Department of Urology, Union Hospital, Huazhong University of Science and Technology, Wuhan, China

**Keywords:** ABMR, kidney transplantation, PE, SARS-CoV-2, IVIG

## Abstract

**Background:**

Due to its high mutation rate, severe acute respiratory syndrome coronavirus 2 (SARS-CoV-2) has recurrently emerged worldwide in recent years, leading to an increased incidence of rejection following kidney transplantation and a worsened prognosis for recipients. The management of the concomitant occurrence of SARS-CoV-2 infection and rejection in kidney transplant recipients poses significant challenges, with limited available experience on this topic. This study presents a case report highlighting the simultaneous manifestation of severe corona virus disease 2019 (COVID-19) pneumonia and acute antibody-mediated rejection (ABMR) during the early post-transplantation period.

**Methods:**

The recipient underwent the renal transplantation from a deceased donor after brain death and received comprehensive management including antiviral therapy, adjustment of immunosuppressive medications, and relevant supportive care during the course of SARS-CoV-2 infection. In the overlapping period of severe COVID-19 pneumonia and ABMR, we implemented plasma exchange (PE) combined with intravenous immunoglobulin (IVIG) and rituximab treatment, while closely monitoring infection-related indicators and elucidate the impact of PE on SARS-CoV-2 antibodies.

**Results:**

The administration of PE did not significantly impact the level of SARS-CoV-2 IgG antibody. Meanwhile, the combination of PE, IVIG, and rituximab treatment effectively reversed ABMR without exacerbating SARS-CoV-2 infection.

**Conclusion:**

The timely administration of antiviral and anti-rejection therapies in the early stage of renal transplant recipient can lead to favorable outcome in case of SARS-CoV-2 infection and concurrent ABMR.

## Introduction

Since the emergence of severe acute respiratory syndrome coronavirus 2 (SARS-CoV-2), a highly mutable virus, it has caused periodic global epidemics in recent years. The disease compromises the human immune system, resulting in an increased susceptibility to other respiratory infections such as adenovirus and influenza, which also exacerbates the impact of Corona virus disease 2019 (COVID-19) on kidney transplant recipients. Scholars have discovered that COVID-19 has resulted in a decrease in adherence and dosage of oral immunosuppressive medications among post-kidney transplant recipients, consequently elevating the risk of rejection (1). Furthermore, SARS-CoV-2 infection can induce acute kidney injury and impact the long-term functionality of the transplanted kidney (2, 3).

Antibody-mediated rejection (ABMR) presents a significant challenge in the field of organ transplantation. In contrast to T-cell mediated rejection (TCMR), ABMR is characterized by B lymphocyte activation and specific antibody production. The treatment for ABMR involves plasma exchange (PE)/immunoadsorption and intravenous immunoglobulin (IVIG) administration to eliminate circulating antibodies; however, the efficacy of these interventions remains limited (4).

Our center has recently encountered a unique case involving a patient who developed concurrent SARS-CoV-2 infection and ABMR in the recent term after kidney transplantation. Because the lack of established protocols for treating ABMR following SARS-CoV-2 infection, we initiated PE and other relevant treatments to prevent graft failure and recorded the impact to the SARS-CoV-2 infection. This case report offers valuable insights of management of concurrent ABMR and SARS-CoV-2 infection.

## Case presentation

The donor, a 47-year-old male with a height of 176 cm and weight of 76 kg, succumbed to brainstem hemorrhage without any history of diabetes or hypertension. The serum creatinine (SCr) level was measured at 80.7 μmol/L prior to kidney procurement. The recipient, a 36-year-old male measuring 173 cm in height and weighing 76 kg, received the left kidney for transplantation. The right kidney was allocated to another patient within our center for transplantation. Renal grafts were donated to the Red Cross Society and allocated to our center by the China Organ Transplant Response System. The procedures were in compliance with the national program of organ donation in China, the Helsinki Congress as well as the Declaration of Istanbul.

The warm ischemia time lasted for only 2 min, while the cold ischemia time extended to 3 h. There was a mismatch of human leukocyte antigens (HLA) by 4/6. The recipient’s diagnosis of IgA nephropathy followed the discovery of proteinuria 15 years ago through renal biopsy. Due to deteriorating renal function, he commenced hemodialysis seven years ago and currently undergoes treatment three times per week. Prior to kidney transplantation, there is no record of blood transfusion or SARS-CoV-2 vaccination for the recipient. Both the donor and recipient tested negative for SARS-CoV-2 nucleic acid before organ procurement.

The recipient received 1 mg/kg of rabbit anti-human thymocyte immunoglobulin (rATG) at day 0, followed by the same dosage at day 1 and half dosage at day 2. Daily 500 mg of methylprednisolone was given intravenously at day 0, 1 and 2, followed by oral prednisone tapering the dosage. Triple therapy immunosuppressants with tacrolimus, mycophenolate mofetil (MMF) and corticosteroids were maintained, and target trough concentration level for tacrolimus was 5–15 ng/mL. Other specific methodologies are detailed in Supplementary File 1.

**FIGURE 1:**
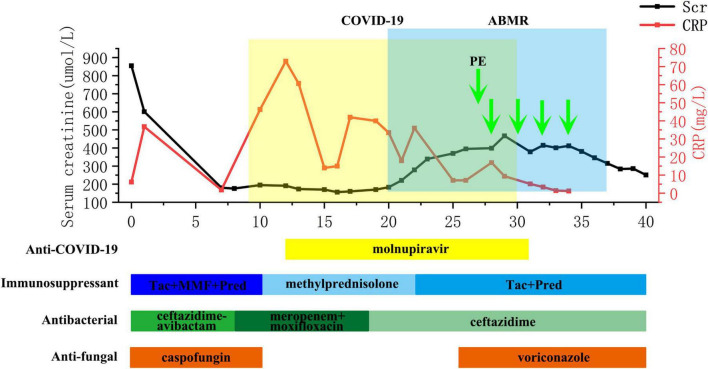
Flow chart of concurrent severe COVID-19 pneumonia and ABMR course of the recipient. ABMR, antibody-mediated rejection; CRP, C-reactive protein; MMF, mycophenolate mofetil; PE, plasma exchange; SCr, serum creatinine; Tac, tacrolimus.

**FIGURE 2:**
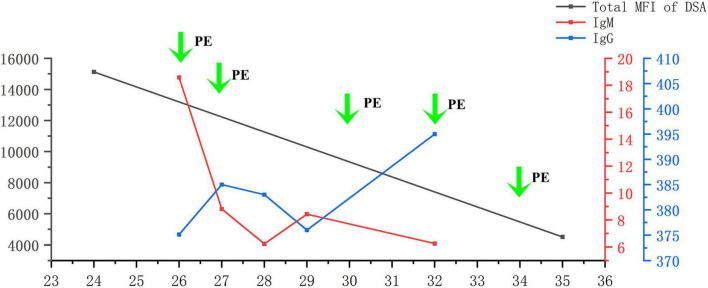
Levels of DSA, SARS-CoV-2 IgG, and SARS-CoV-2 IgM during PE. DSA, donor-specific antibody; PE, plasma exchange.

**FIGURE 3:**
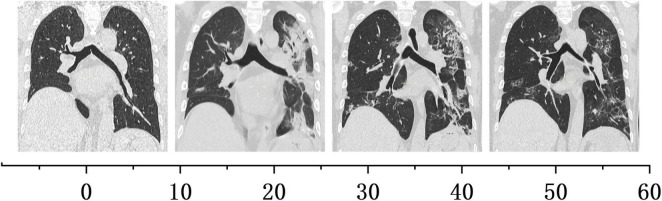
Computed tomography images of lung after kidney transplantation.

The flow chart illustrating the complete disease progression is depicted in Figure 1. The recipient developed fever, cough, and decreased oxygen saturation (SpO2 88% without supplemental oxygen) on day 9 post-transplant. The cycle threshold of SARS-CoV-2 nucleic acid was below 30, indicating a high viral load, while lung computed tomography scans revealed extensive ground glass opacities. In response, we administered antiviral treatment with molnupiravir, adjusted antibiotic therapy, and gradually suspended MMF and tacrolimus, while maintaining immunosuppression with prednisolone 40 mg daily. In the subsequent week, there was a gradual decline in the patient’s inflammatory markers and SARS-CoV-2 nucleic acid levels, while body temperature was partially controlled but failed to normalize. To further characterize the respiratory infection type, fiberoptic bronchoscopy was performed on postoperative day 14, obtaining an alveolar-rinse sample for next generation sequencing which exclusively identified the presence of SARS-CoV-2 sequence. However, on day 19 post-transplant, the patient’s body temperature remained abnormal and there was a significant increase in SCr level. Despite maintaining a urine output of approximately 3000 ml per day, the recipient experienced a substantial weight gain. The patient underwent two hemodialysis sessions in order to maintain water and electrolyte balance. The detection of HLA antibodies revealed the presence of denovo donor-specific antibody (dnDSA), with positive results observed in 7 out of 18 sites (A/B/DRB1/DRB3/DPB1). The cumulative MFI value of DSA was measured at 15127, while SCr increased to 370 μmol/L from the lowest 156 μmol/L. Tacrolimus was reintroduced and treatment for ABMR was initiated on day 26, which included five PE sessions, IVIG administration at a dose of 100 mg/kg after PE, and rituximab administration at a dose of 300 mg after the last PE. Considering that the recipient’s nucleic acid test for SARS-CoV-2 continued to be positive, we regularly monitored SARS-CoV-2 antibodies (IgM, IgG) before and after each PE and conducted regular etiological and imaging examinations (Figures 2, 3). After undergoing five sessions of PE, the number of dnDSA sites decreased from 7 to 3 (3/18, B/DRB1/DPB1), and the total MFI of DSA decreased to 4508. The patient’s renal function exhibited significant improvement as his body temperature returned to normal. By monitoring indicators related to SARS-CoV-2, we observed that anti-ABMR treatment did not result in a rebound in white blood cell count and C-reactive protein (CRP) levels. The presence of SARS-CoV-2 nucleic acid became completely negative after the third PE. Simultaneously, there was no significant change in the level of SARS-CoV-2 IgG, while the level of IgM decreased by half compared to pre-treatment level.

One week later, the patient developed severe oral ulcers and cytomegalovirus (CMV) viremia; therefore, we adjusted his immunosuppressive regimen accordingly and initiated ganciclovir intravenously. The serum CMV-DNA level returned to normal, leading to the discontinuation of intravenous ganciclovir and a switch to oral valganciclovir for ongoing therapy and long-term prophylaxis. At discharge on day 55, the patient’s SCr level was 231 μmol/L.

After a 4-month follow-up period post-discharge, the patient’s bilateral lung infection foci were completely resolved, with negative DSA results and the SCr level around 230 μmol/L.

## Discussion

This article presents, for the first time, the management of concurrent severe COVID-19 pneumonia and ABMR in the early post-kidney transplant period. Despite the risk of exacerbating SARS-CoV-2 infection, we employed the treatment regimen involving PE, IVIG, and rituximab to eliminate dnDSA while the patient still tested positive for SARS-CoV-2 nucleic acid. Following this treatment, ABMR was effectively controlled, with a decrease in SARS-CoV-2 IgM level and stabilization of IgG level. Subsequently, the patient was successfully discharged from the hospital.

Kidney transplant recipients are at an increased risk of developing severe COVID-19 pneumonia and experiencing higher mortality rates (5). A multicenter study conducted in France identified several risk factors associated with mortality following SARS-CoV-2 infection, including elderly recipients (>60 years), hypertension, cardiovascular disease, diabetes, elevated post-infection CRP levels (>60 mg/L), lymphopenia, and increased postoperative SCr levels (>115 μmol/L) (6). In our case report, the recipient presented with hypertension, incomplete graft function recovery prior to infection, and a post-infection CRP level exceeding 60 mg/l, indicative of severe COVID-19 infection. There is now substantial evidence indicating the favorable efficacy of molnupiravir in the treatment and prevention of COVID-19. Despite its lower effectiveness compared to other antiviral drugs like nematavir/ritonavir, molnupiravir is widely utilized among transplant recipients due to its non-interference with tacrolimus concentration (7, 8). In this case, the estimated glomerular filtration rate was below 30 ml/min at the time of COVID-19 diagnosis, and significant reductions in inflammatory factors were observed following molnupiravir treatment. Building upon our previous experience (9), we administered an extended course of molnupiravir, resulting in a negative SARS-CoV-2 nucleic acid test on day 17 of treatment.

The impact of COVID-19 on the immune system varies across different stages of the pandemic. During the acute phase of infection, SARS-CoV-2 enhances patients’ susceptibility to co-infections, such as influenza virus, by inhibiting interferon signaling pathways and impairing T-cell functionality (10). In the post-pandemic era, severe COVID-19 pneumonia can result in prolonged immune dysregulation, characterized by a sustained elevation in IL-6 levels and a reduction in regulatory T cell counts. This immune imbalance may lead to the reactivation of latent viruses (such as CMV) or the onset of autoimmune disorders (11, 12). For kidney transplant recipients, prolonged chronic immunosuppression significantly exacerbates this risk. Notably, in this case, CMV emerged following treatment for ABMR, which may be associated with the overproduction of inflammatory factors induced by COVID-19 and the resultant excessive immunosuppression. On the other hand, SARS-CoV-2 infection has been associated with an increased incidence of rejection in transplant recipients. When immunosuppression is temporarily reduced, the HLA epitope mismatch load between the donor and recipient influences the risk of dnDSA. Castrezana-Lopez et al. reported that among 47 renal transplant recipients, 14 (30%) developed new HLA antibodies following a period of reduced immunosuppression, with 4 (9%) developing dnDSA (1). DnDSA are the primary drivers of ABMR, implying that COVID-19 may indirectly elevate the risk of ABMR. Regarding vaccine application, while there have been a few reports suggesting that SARS-CoV-2 mRNA vaccines may induce dnDSA in kidney transplant recipients, most evidence indicates that their administration can effectively reduce infection rates and severity among such individuals (13).

This case highlights two unique aspects compared to prior reports. First, while most studies describe rejection episodes occurring >1 month post-transplant and independently of COVID-19 (14), our patient developed ABMR concurrent with severe COVID-19 pneumonia within 30 days post-surgery. Second, unlike T cell-mediated rejection (TCMR), which responds well to steroid pulses or rATG, ABMR lacks standardized therapies and relies on modalities like PE, IVIG, and rituximab with limited success (4, 15). Notably, Sherwood et al. reported TCMR resolution post-COVID-19 using steroids (16), whereas Nourie et al. observed chronic ABMR development post-infection despite IVIG therapy (17), suggesting delayed intervention may worsen outcomes. The early dnDSA emergence here (vs. pre-existing antibodies in typical ABMR) and our use of rATG induction (vs. basiliximab in other cohorts) further distinguish this case (Supplementary File 2). Collectively, these findings underscore that ABMR overlapping with active COVID-19 necessitates early intervention despite infection risks, as timely PE/IVIG/rituximab can prevent irreversible graft injury.

The management of ABMR has always posed a challenging issue following renal transplantation. Early ABMR is predominantly associated with preformed donor-specific antibody (pfDSA), which may result in short-term graft dysfunction (4). However, in this particular case, we conducted HLA antibody testing on the day of surgery and did not detect any DSA. Therefore, we considered it to be dnDSA rather than pfDSA. Due to prior rATG induction therapy and unresolved SARS-CoV-2 infection in the recipient, steroid pulse therapy was withheld and the dose of rituximab was reduced. It is noteworthy that within 30 days post-transplantation, the patient developed dnDSA without evidence of pfDSA presence, leading to rejection, which deviated from most scholarly reports on this matter. We consider that several factors may have contributed to this outcome: (1) Prolonged duration for reduction and withdrawal of immunosuppressive agents (14); (2) Generation of donor-specific memory B lymphocytes during dialysis (15); (3) COVID-19-induced inflammatory cytokine storm causing damage to donor kidney cells, thereby increasing exposure to target antigens and triggering an early anti-donor immune response (18); (4) Possible presence of non-HLA antibodies in the patient (19).

PE can effectively decrease the systemic drug concentration of various medications, including immunosuppressants, cardiovascular drugs, and anti-infective agents. Simultaneously, it facilitates the elimination of macromolecular substances larger than 150 kDa from circulation, such as specific IgG, IgM, and DSA. Wang et al. observed that the initial PE exhibited optimal clearance efficiency in rescuing patients with amphotericin B liposome poisoning; however, subsequent PE did not alter the metabolic process of amphotericin B liposome within the body due to potential drug redistribution and equilibrium (20). In this case, we conducted a total of five PE, each involving an exchange volume of 2000 ml. As a result, IgM antibody level in the recipient decreased following the procedure; however, no significant decrease was noted in IgG level. We consider that this phenomenon can be partially attributed to immunoglobulin rebalancing induced by PE. Additionally, we also considered that this could be attributed to the infused plasma component subsequent to PE. The patient underwent voluntary blood donation using plasma. A study conducted in China between 2022 and 2023 on voluntary blood donors revealed a SARS-CoV-2 antibody detection rate of 49.1%, with the highest detection rate reaching 93.9%. Another study demonstrated that recovered patients maintained a level of SARS-CoV-2 antibodies for over 12 months. After 1 year, more than 50% recovered patients had IgG level greater than 1:160, and over 25% of the population had IgG level greater than 1:320 (21). The aforementioned report elucidates why this patient’s IgG level remained high after PE while his IgM level significantly decreased; we believe that the plasma used for PE contained a substantial amount of SARS-CoV-2-specific IgG antibody, thereby providing protection to the patient.

By reason of the foregoing, this is the first report of concurrent severe COVID-19 pneumonia and ABMR occurring within 30 days post-transplantation. The unique challenges included (1) overlapping risks of infection exacerbation and graft loss, (2) the use of PE/IVIG/rituximab despite active SARS-CoV-2 positivity, and (3) demonstration that PE does not significantly clear SARS-CoV-2 IgG—a critical finding for managing COVID-19 in transplant recipients requiring antibody removal.

Our study has several limitations, including small case number and the relatively shorter follow-up duration. To address these limitations, a comprehensive follow-up study will be conducted for an extended period. Furthermore, in subsequent cases of COVID-19 complicated with ABMR, plasma samples were collected prior to PE for the detection of IgG levels to substantiate our speculation.

## Conclusion

This study presents a rare case of severe COVID-19 pneumonia overlapping with ABMR following kidney transplantation. We observed that effective management of immunosuppressive drugs and antiviral treatment can lead to successful resolution of severe COVID-19 pneumonia even during the initial stages after kidney transplantation. Additionally, we found that within 1 month after renal transplantation, ABMR may also be attributed to dnDSA. However, employing PE along with IVIG and rituximab therapy can significantly improve outcomes and prevent renal graft failure caused by ABMR. Lastly, considering the ongoing recurrent outbreaks of COVID-19, it is important to note that blood donation for PE does not result in substantial clearance of SARS-CoV-2 IgG or exacerbation of SARS-CoV-2 infection.

## Data Availability

The original contributions presented in this study are included in this article/Supplementary material, further inquiries can be directed to the corresponding author.
