# Supplementary material for: Management of concurrent severe COVID-19 pneumonia and antibody-mediated rejection following kidney transplantation: a case report
Source: Front Med (Lausanne). 2025 Mar 13;12:1521785. doi: 10.3389/fmed.2025.1521785 (PMC11966428; doi:10.3389/fmed.2025.1521785)
Supplement: Supplementary file 1 [file Supplementary_file_1.docx]

Supplementary file 1: Perioperative and Postoperative Management

The original protocol detailing postoperative immune induction and immunosuppression has been outlined.

Standardized Postoperative Management

The drainage fluid and urine samples from patients were collected on the first postoperative day for Next-Generation Sequencing (NGS) analysis and culture. These results were integrated with NGS and culture findings obtained during the donor maintenance phase to guide adjustments in the recipients' antibiotic regimens. Postoperatively, for one week, daily monitoring included routine blood tests, blood biochemistry panels, infection markers, inflammatory factor levels, and urine output. The concentrations of immunosuppressive drugs were assessed two to three times per week.

On the first postoperative day, color Doppler ultrasound was conducted to assess for potential vascular complications. A CT scan of the chest, abdomen, and pelvis was performed between the second and third postoperative days to evaluate for any hemorrhage or pulmonary infection in the surgical area. Subsequently, ultrasound and CT scans were repeated based on the patients' clinical manifestations and laboratory findings. Throughout the hospital stay, vital signs including body temperature, blood pressure, heart rate, blood oxygen saturation, blood glucose, and lipid levels were monitored regularly. Patients were instructed to expectorate following daily nebulization therapy and to adhere to respiratory function exercises.

Management of recipients with postoperative fever

The patient had fever after operation, and the inflammatory indicators such as CRP and PCT were routinely reexamined, and the blood and urine routine, blood and urine sputum bacterial and fungal culture were improved. At the same time, routine screening for SARS-CoV-2, influenza, mycoplasma, CMV and other infections was performed, and G, GM and t-spot tests were further improved when necessary.When oxygen saturation decreased, patients underwent repeat lung CT scans. If abdominal infection was suspected based on clinical symptoms, ascites B-ultrasound and CT examinations were conducted. Fiberoptic bronchoscopy or abdominal paracentesis was performed as required. Specimens were collected for culture and NGS to identify the infectious agent. Additionally, we monitored for fever that could be attributed to other causes such as rejection. Therefore, HLA antibody levels, renal function, and inflammatory markers were routinely reevaluated seven days post-operation, with renal allograft biopsy performed if indicated.

Treatment of Infection

In our center, a routine prophylactic regimen of broad-spectrum antibiotics (covering bacteria, fungi, and viruses) was administered postoperatively to prevent infections. Upon identification of multi-drug resistant Klebsiella pneumoniae in the donor's sputum culture, the postoperative antibiotic prophylaxis was escalated to ceftazidime-avibactam. Despite this adjustment, the patient subsequently developed a fever. Given the lack of improvement with empirical anti-infective therapy, the antibiotic regimen was further escalated to meropenem in combination with moxifloxacin. However, subsequent NGS analysis of the patient's bronchoscopic sputum sample revealed only the presence of SARS-CoV-2. Consequently, we de-escalated the antibacterial therapy to ceftazidime.

Monolavir is the first choice for the treatment of COVID-19 in our center. We routinely monitor SARS-CoV-2 RNA levels and antibody titers to assess infection control efficacy and adjust the immunosuppressive regimen as needed based on patient status. Adjuvant therapies for severe COVID-19 pneumonia include respiratory support, anticoagulation, anti-inflammatory measures, prone positioning ventilation, nutritional and metabolic support, respiratory rehabilitation, and symptomatic management.

SARS-CoV-2 antibody and donor-specific antibody detection

Enzyme-linked immunosorbent assay (ELISA) was used to detect the IgM/IgG of SARS-CoV-2 binding antibodies. < 10AU/ML was defined as non-reactive, and ≥10AU/ml was defined as reactive.

Donor-specific antibodies (DSA) were detected by Luminex single antigen-bead assay (SAB) (MFI value reference range: negative < 750, weakly positive 750-3999, moderately positive 4000-10000, strongly positive > 10000).

Hemodialysis and plasmapheresis

In accordance with the patient's postoperative condition, hemodialysis and plasma exchange therapies were employed. The specific parameters are detailed as follows:

Hemodialysis

Machine:4008 S Version V10, Fresenius Medical Care, German

Fiber dialyzer: REXEED-18UC, AsahiKASEI

Vascular access: central venous catheter or internal arteriovenous fistula

Blood flow:230~250ml/min

Dialysis frequency: 4 hours a time

Anticoagulation: low molecular weight heparin or Nafamostat Mesilate

Plasmapheresis

Machine: Diapact CRRT, B.Braun Avitum, German

Plasma separator: Plasmaflo OP-08W

Secondary filter: Cascadeflo EC-20W

Vascular access: central venous catheter or internal arteriovenous fistula

Blood flow:150ml/min

Plasma flow:25ml/min

Anticoagulation: low molecular weight heparin or Nafamostat Mesilate

Each session exchanged 2000 mL plasma replacement. IVIG (100 mg/kg) was administered post-PE, followed by rituximab 300 mg after the final PE.

Postoperative follow-up management

Following discharge, blood routine tests, biochemical blood analyses, urine routine tests, and blood drug concentration monitoring were conducted regularly in accordance with the guidelines. A follow-up lung CT scan was performed one month post-discharge. DSA was re-evaluated at 2, 3, and 7 months post-operation, with results turning negative by the 7-month. Additionally, regular monitoring of CMV, EBV, and BKV was conducted.
