# Supplementary material for: Management of concurrent severe COVID-19 pneumonia and antibody-mediated rejection following kidney transplantation: a case report
Source: Front Med (Lausanne). 2025 Mar 13;12:1521785. doi: 10.3389/fmed.2025.1521785 (PMC11966428; doi:10.3389/fmed.2025.1521785)
Supplement: Supplementary file 2 [file Supplementary_file_2.docx]

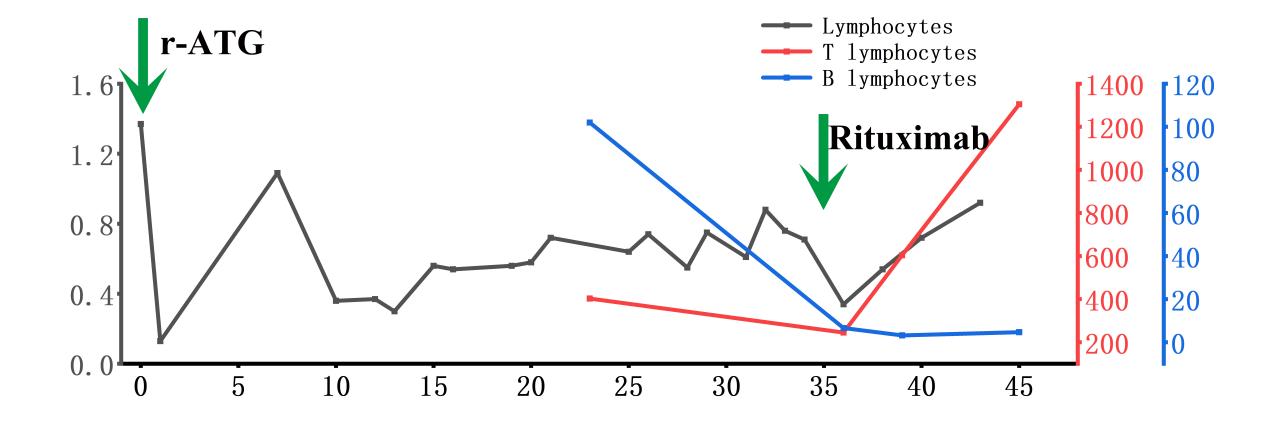


Figure S1 Alterations in lymphocyte levels following renal transplantation. rATG, rabbit anti-human thymocyte globulin
